# Application of preoperative ultrasound features combined with clinical factors in predicting HER2-positive subtype (non-luminal) breast cancer

**DOI:** 10.1186/s12880-021-00714-0

**Published:** 2021-12-02

**Authors:** Jin Zhou, An-qi Jin, Shi-chong Zhou, Jia-wei Li, Wen-xiang Zhi, Yun-xia Huang, Qian Zhu, Lang Qian, Jiong Wu, Cai Chang

**Affiliations:** 1 Department of Ultrasound, First Floor, Building 3, Fudan University Shanghai Cancer Center, No. 270 Dong’an Road, Xuhui District, Shanghai, China; 2 Department of Breast Surgery, Fudan University Shanghai Cancer Center, Shanghai, China; 3 Department of Oncology, Shanghai Medical College, Fudan University, Shanghai, China

**Keywords:** Breast cancer, Estrogen receptor, Progesterone receptor, Human epidermal growth factor receptor-2, Ultrasound

## Abstract

**Background:**

Human epidermal growth factor receptor2+ subtype breast cancer has a high degree of malignancy and a poor prognosis. The aim of this study is to develop a prediction model for the human epidermal growth factor receptor2+ subtype (non-luminal) of breast cancer based on the clinical and ultrasound features related with estrogen receptor, progesterone receptor, and human epidermal growth factor receptor2.

**Methods:**

We collected clinical data and reviewed preoperative ultrasound images of enrolled breast cancers from September 2017 to August 2020. We divided the data into in three groups as follows. Group I: estrogen receptor ± , Group II: progesterone receptor ± and Group III: human epidermal growth factor receptor2 ± . Univariate and multivariate logistic regression analyses were used to analyze the clinical and ultrasound features related with biomarkers among these groups. A model to predict human epidermal growth factor receptor2+ subtype was then developed based on the results of multivariate regression analyses, and the efficacy was evaluated using the area under receiver operating characteristic curve, accuracy, sensitivity, specificity.

**Results:**

The human epidermal growth factor receptor2+ subtype accounted for 138 cases (11.8%) in the training set and 51 cases (10.1%) in the test set. In the multivariate regression analysis, age ≤ 50 years was an independent predictor of progesterone receptor + (p = 0.007), and posterior enhancement was a negative predictor of progesterone receptor + (p = 0.013) in Group II; palpable axillary lymph node, round, irregular shape and calcifications were independent predictors of the positivity for human epidermal growth factor receptor-2 in Group III (p = 0.001, p = 0.007, p = 0.010, p < 0.001, respectively). In Group I, shape was the only factor related to estrogen receptor status in the univariate analysis (p < 0.05). The area under receiver operating characteristic curve, accuracy, sensitivity, specificity of the model to predict human epidermal growth factor receptor2+ subtype breast cancer was 0.697, 60.14%, 72.46%, 58.49% and 0.725, 72.06%, 64.71%, 72.89% in the training and test sets, respectively.

**Conclusions:**

Our study established a model to predict the human epidermal growth factor receptor2-positive subtype with moderate performance. And the results demonstrated that clinical and ultrasound features were significantly associated with biomarkers.

## Introduction

Breast cancer is a highly heterogenous tumor that has recently become the most common malignant tumor worldwide [1, 2]. The 2013 St. Gallen Consensus classified breast cancer into five subtypes according to the biomarker expression (estrogen receptor [ER], progesterone receptor [PR], human epidermal growth factor receptor-2 [HER2], and Ki67) evaluated using immunohistochemistry (IHC) [3]. Different breast cancer subtypes and biomarker expression of breast cancer are important prognostic factors [4, 5].

HER2+ breast cancer accounts for about 15– 20% of all breast cancers [6]. HER2+ subtype (non-luminal) breast cancer is defined as ER-, PR-, HER2+, and has a high degree of malignancy and a poor prognosis, with a heterogeneous clinical and biological presentation. Hereafter, HER2+ subtype refers to HER2+ subtype (non-luminal). Chromosome 17 polyploidy, spatial and temporal heterogeneity of tumors lead to inaccurate assessment of HER2 status [7]. And HER2 score 2 + on IHC require additional fluorescent in-situ hybridization (FISH) or chromogenic in-situ hybridization (CISH) testing to determine their status [7, 8]. They adversely affect the diagnosis and treatment of HER2+ breast cancer. Currently, the diagnosis of breast cancer subtypes and biomarkers of breast cancer requires preoperative core-needle or postoperative pathology, which is an invasive and time-consuming process. If these could be obtained preoperatively and noninvasively, it would make the treatment process more timely, effective and precise.

In Asian women, ultrasound (US) is the method of choice for screening breast lesions since they have denser breast tissue and are relatively younger at the time of diagnosis [9–12]. Previous studies have found correlations between biomarkers (ER, PR, and HER2) and US features [13–15]. Xu et al. [15] found that the longest/shortest size ratio (> 1), spiculate margin, and echo halo were related to ER and PR positivity. Additionally, Liu et al. [14] suggested that HER2 positivity was related to tumor blood supply and microcalcification. However, these studies utilized a relatively small number of cases and have correlated the relevant features directly with biomarkers (e.g., ER ± , regardless of the status of PR, HER2). Thus, all three biomarkers had an impact on the US findings of breast cancer. When two biomarkers were known to be in the same status, studying the relationship between US features and one biomarker may be a feasible approach.

Previous studies [2, 16, 17] revealed a correlation between the HER2+ subtype and US features, such as posterior enhancement or calcifications. However, many studies on conventional US features and breast cancer subtypes have been limited to correlation exploration [16–19], and fewer studies have built predictive models. In contrast to previous studies [2, 16, 17] that directly correlated breast cancer subtypes with relevant features, we aimed to build a predictive model for the HER2+ subtype using relevant features of three biomarkers and evaluate its performance.

The purpose of this study was to evaluate the associated clinical and US features of ER, PR, and HER2 when two biomarkers were in known same status, and then develop a predictive model for the HER2+ subtype. To the best of our knowledge, this study is the first to identify clinical and US features associated with ER, PR, and HER-2 status when two biomarkers were known to be in the same status.

## Methods

### Study population

This study included patients who underwent preoperative breast US in projects funded by the National Natural Science Foundation from September 2017 to August 2020. Patients with the following characteristics were included: (1) evident lesions on preoperative US images, and multiple US images of breast tumors; (2) breast cancers diagnosed by core needle biopsy or surgical pathology; and (3) the absence of treatment, prior to US. Patients with the following characteristics were excluded: (1) any treatment, such as radiotherapy, before US examination; (2) invisible or obscure lesions on the US examinations; or (3) incomplete clinical data.

This retrospective study was approved by the institutional ethics committee of our center. The requirement for informed consent was waived due to the retrospective nature of the study. We randomized the enrolled data into a training set (1169 cases) and a test set (501 cases) by a 7:3 ratio.

As mentioned previously, no article considered the possible interaction of biomarkers on the ultrasound feature of breast cancers. Thus, our study made two of the three markers in identical status between two groups to analyze whether the remaining markers have a relationship with ultrasound features. Since no case presented ER− and PR+ and only 55 cases were ER+, PR−, HER2+, only the following classification of cases could be studied in this study. To facilitate follow-up studies and promote understanding, the cases were grouped as follows, Group I: ER+ vs ER− (PR and HER2 negative), Group II: PR+ vs PR− (ER+, HER2−), and Group III: HER2+ vs HER2− (ER and PR negative).

### Pathology and immunohistochemistry analysis

Data regarding the age, BMI (body mass index), menopause, palpable axillary lymph node (ALN), breast cancer family history, US ALN, pathological type, histological grade, and ER, PR, HER2 status were collected from the medical record system. The positivity of ER or PR is defined as ≥ 1% on IHC staining [3]. The positivity of HER2 is defined by any of following test results: (1) IHC 3 + , or complete and strong member staining of > 30% of invasive cancer cells; (2) FISH measurement of HER2/CEP17 ratio of > 2.2/2.0; and (3) CISH of a HER2 gene copy number of > 6.0 signals per nucleus [8].

### US images assessment

Most breast US images were obtained using the SuperSonica Aixplorer US scanner (SuperSonic Imagine S.A., Aix-en-Provence, France) equipped with a 7–15 MHz linear array transducer. Other breast US images were obtained using the Mindray Resona 5S US scanner (Shenzhen Mindray Bio-Medical Electronics Co., Ltd., Shenzhen, China) equipped with a 5–14 MHz linear array transducer. The imaging acquisition standards were as follows: 12 conventional US images were captured starting with the largest cross-section of the tumor at equal intervals in a 180° clockwise range. Suspicious breast lesions were measured at the maximal diameter on US images.

The US features were assessed according to the Breast Imaging-Reporting and Data System [20, 21], including shape, orientation, margins, boundary, echo pattern, calcification, and posterior acoustic features. Additionally, vascularity was assessed according to Adler's index (0, I, II, or III) [22]. All US images were reviewed by two US specialists who were blinded to the patients’ pathological results. If the two reviewers disagreed, a consensus was reached after their discussion.

### Statistical analysis

Data analysis was performed using SPSS version 20 (IBM Corp, Armonk, NY, USA). The data were randomly divided into the training and test sets using random numbers. Normally distributed data, assessed using the Kolmogorov–Smirnov test, were expressed as mean ± standard deviation. And non-normally distributed data were expressed as median with interquartile range. Categorical variables were analyzed using the Chi-square or Fisher’s exact test. Multivariate logistic regression analysis (backward stepwise) was used to study the association between clinical and US features and the three biomarkers. The model was built using R software (version 4.1.0, R Foundation for Statistical Computing, Vienna, Austria) based on the independent predictors from the multivariate regression analysis, and the diagnostic efficacy of the model was evaluated using the area under the receiver operating characteristic curve (AUC), accuracy (ACC), sensitivity (SENS) and specificity (SPEC). Statistical significance was set at P < 0.05.

## Results

### Clinical and pathological characteristics

**Fig. 1:**
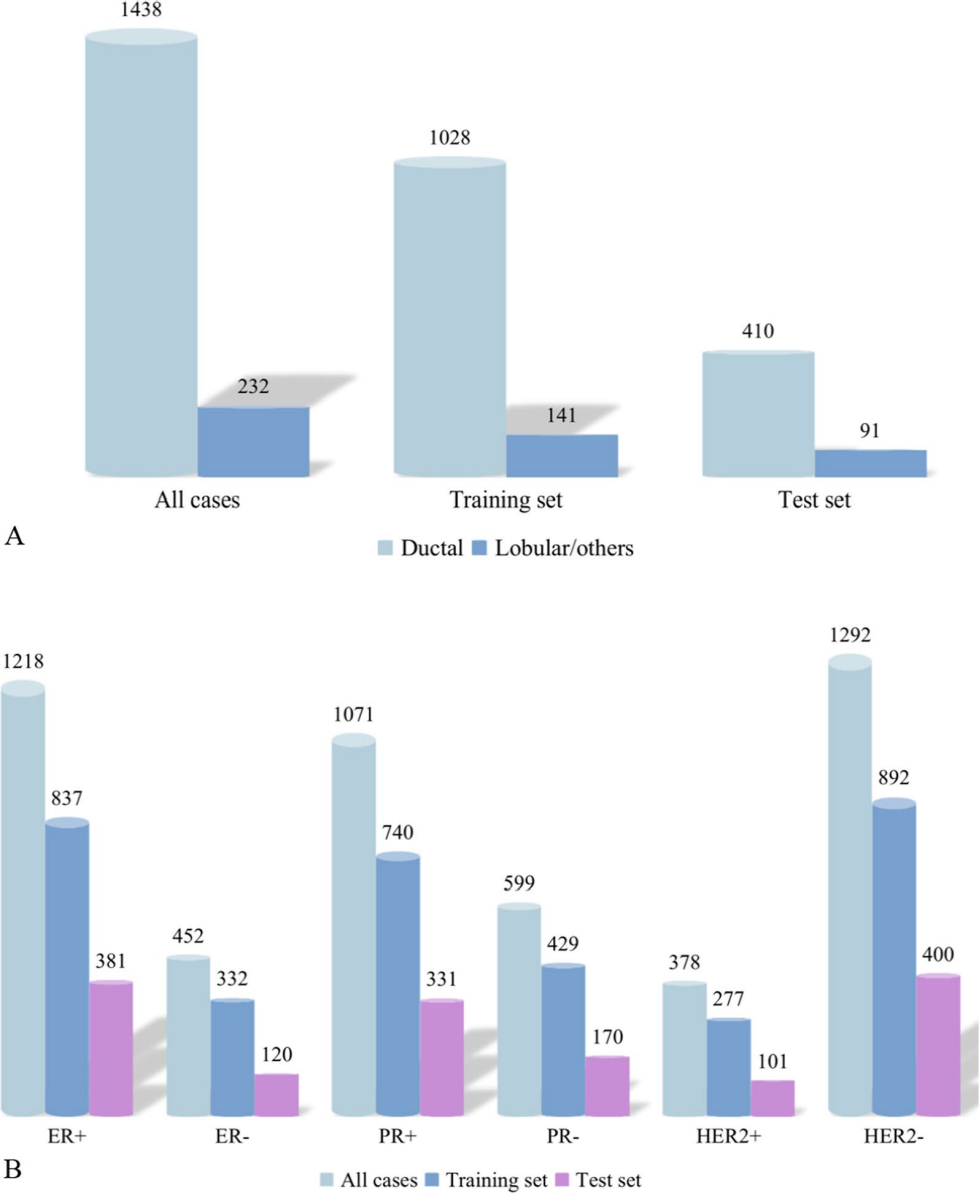
The distribution of pathological types (**A**) and ER, PR, HER2 (**B**) status among all cases, training and test sets. In this paper, the pathological types of breast cancer were categorized into two types: ductal carcinoma, lobular carcinoma and other types. And the distribution and number of cases in the three groups are shown in (**A**). **B** shows the distribution and number of cases of ER, PR and HER2 expression status in the three groups. *ER* estrogen receptor, *PR* progesterone receptor, *HER2* human epidermal growth factor receptor-2

The study enrolled 1670 breast cancer cases (1662 female patients) with a mean age of 53.1 years (range 22–95 years) and mean tumor size of 21.0 mm (range 6–68 mm). The training set had a mean age of 53 years (range 22–95 years) and mean tumor size of 20 mm (range 5–68 mm), while the test set had a mean age of 53 years (range 28–87 years) and mean tumor size of 20 mm (range 6–56 mm). The two sets were not statistically different at the baseline of clinical and US features (excluding calcifications). All details are shown in Table 1 and Fig. 1.

**Table 1:** Clinicopathological characteristics and baseline of all cases, training set and test set

Characteristics	All cases (N = 1670)	Training set (N = 1169)	Test set (N = 501)	*P* value
Age (year)	53.1 [45, 61]	53 [45,61]	53 [46,61]	0.604
tumor size (mm)	21.0 [15, 26]	20.0 [15, 26]	20.0 [15, 26]	0.393
BMI value	23.3 [21.4, 25.4]	23.3 [21.5, 25.4]	23.23 [21.2, 25.1]	0.335
Menopause				0.724
No	769 (46.0)	535 (45.8)	234 (46.7)	
Yes	901 (54.0)	634 (54.2)	267 (53.3)	
Breast cancer family history				0.566
No	1610 (96.4)	1125 (96.2)	485 (96.8)	
Yes	60 (3.6)	44 (3.8)	16 (3.2)	
Palpable ALN				0.534
No	1348 (80.7)	939 (80.3)	409 (81.6)	
Yes	322 (19.3)	230 (19.7)	92 (18.4)	
US ALN				
No	1031 (61.7)	712 (60.9)	319 (63.7)	0.287
Yes	639 (38.3)	457 (39.1)	182 (36.3)	
Shape				
Oval	90 (5.4)	67 (5.7)	23 (4.6)	0.259
Round	62 (3.7)	48 (4.1)	14 (2.8)	
Irregular	1518 (90.9)	1054 (90.2)	464 (92.6)	
Orientation				0.860
Parallel	1048 (62.8)	732 (62.6)	316 (63.1)	
Not parallel	622 (37.2)	437 (37.4)	185 (36.9)	
Margin				0.690
Circumscribed	37 (2.2)	27 (2.3)	10 (2.0)	
Not circumscribed	1633 (97.8)	1142 (97.7)	491 (98.0)	
Boundary				0.168
Abrupt	1068 (64.0)	760 (65.0)	308 (61.5)	
Halo	602 (36.0)	409 (35.0)	193 (38.5)	
Echo pattern				0.945
Hypoechoic	1483 (88.8)	1041 (89.1)	442 (88.2)	
Isoechoic	57 (3.4)	40 (3.4)	17 (3.4)	
Complex	103 (6.2)	70 (6.0)	33 (6.6)	
Hyperechoic	27 (1.6)	18 (1.5)	9 (1.8)	
Posterior acoustic features				0.379
No	504 (30.2)	350 (30.0)	154 (30.7)	
Enhancement	560 (33.5)	393 (33.6)	167 (33.4)	
Shadowing	539 (32.3)	385 (32.9)	154 (30.7)	
Combined	67 (4.0)	41 (3.5)	26 (5.2)	
Calcifications				0.010
No	826 (49.5)	554 (47.4)	272 (54.3)	
Yes	844 (50.5)	615 (52.6)	229 (45.7)	
Vascular degree				0.973
0	226 (13.5)	160 (13.7)	66 (13.2)	
I	275 (16.5)	190 (16.2)	85 (17.0)	
II	766 (45.9)	535 (45.8)	231 (46.1)	
III	403 (24.1)	284 (24.3)	119 (23.7)	

*BMI* body mass index, *ALN* axillary lymph node, *US* ultrasound

### Relationship among biomarkers, clinical and US features in group I-III from the training set

The following groups were formed as previously described, Group I: ER + vs ER- (PR and HER2 negative), Group II: PR+ vs PR− (ER+, HER2−), and Group III: HER2+ vs HER2− (ER and PR negative).

In Group I, shape was the only factor related to ER status in the univariate analysis (p < 0.05). Oval shape was more frequent in ER-; additionally, older patients (age > 50 years) were more likely to express ER + (Table 2). No multivariate regression analysis was performed because there was only one significant factor in the univariate analysis.

**Table 2:** ER and clinical and ultrasound features in Group I from the training set

Clinical and ultrasound features	Group I		Univariate analysis	
	ER− (PR−, HER2−) (N = 194)	ER+ (PR−, HER2−) (N = 62)	*X*^*2*^ value	P value
Age (year)			3.719	0.054
≤ 50	83 (42.8)	18 (29.0)		
> 50	111 (57.2)	44 (71.0)		
Palpable ALN			0.818	0.366
No	149 (76.8)	51 (82.3)		
Yes	45 (23.2)	11 (17.7)		
US ALN			0.979	0.323
No	105 (54.1)	38 (61.3)		
Yes	89 (45.9)	24 (38.7)		
Breast cancer family history			0.391	0.534
No	184 (94.8)	60 (96.8)		
Yes	10 (5.2)	2 (3.2)		
Menopause			2.374	0.123
No	84 (43.3)	20 (32.3)		
Yes	110 (56.7)	42 (67.7)		
BMI (kg/m^2^)			2.979	0.225
< 18	3 (1.5)	3 (4.8)		
18–24	119 (61.4)	33 (53.2)		
> 24	72 (37.1)	26 (42.0)		
Tumor size (mm)			1.958	0.162
≤ 20	29 (14.9)	14 (22.6)		
> 20	165 (85.1)	48 (77.4)		
Shape			8.255	0.016*
Oval	28 (14.4)	1 (1.6)		
Round	7 (3.6)	4 (6.5)		
Irregular	159 (82.0)	57 (91.9)		
Orientation			2.049	0.152
Parallel	126 (64.9)	34 (54.8)		
Not parallel	68 (35.1)	28 (45.2)		
Margin			0.191	0.662
Circumscribed	5 (2.6)	1 (1.6)		
Not circumscribed	189 (97.4)	61 (98.4)		
Boundary			3.434	0.064
Abrupt	140 (72.2)	37 (59.7)		
Halo	54 (27.8)	25 (40.3)		
Echo pattern			2.338	0.505
Hypoechoic	175 (90.2)	59 (95.2)		
Isoechoic	2 (1.0)	1 (1.6)		
Complex	15 (7.8)	2 (3.2)		
Hyperechoic	2 (1.0)	0 (0.0)		
Posterior acoustic features			6.378	0.095
No	47 (24.2)	14 (22.6)		
Enhancement	106 (54.7)	26 (41.9)		
Shadowing	34 (17.5)	20 (32.3)		
Combined	7 (3.6)	2 (3.2)		
Calcifications			0.304	0.581
No	111 (57.2)	33 (53.2)		
Yes	83 (42.8)	29 (46.8)		
Vascular degree			0.174	0.986
0	27 (13.9)	9 (14.5)		
I	25 (12.9)	9 (14.5)		
II	86 (44.3)	27 (43.6)		
III	56 (28.9)	17 (27.4)		

*ER* estrogen receptor, *PR* progesterone receptor, *HER2* human epidermal growth factor receptor-2, *BMI* body mass index, *ALN* axillary lymph node, *US* ultrasound

In Group II, age, menopause status and posterior acoustic features were related to PR status in the univariate analysis (p < 0.05). Age ≤ 50 years was an independent predictor of PR+ (OR 2.204, 95% CI 1.238–3.924, p = 0.007), and younger patients were 2.204 more likely than older patients to express PR+; additionally, posterior enhancement was a negative predictor of PR+ (OR 0.418, 95% CI 0.211–0.830, p = 0.013), and tumors with posterior enhancement were 0.418 times more likely to exhibit PR+ than tumors with no change in posterior echogenicity (Table 3).

**Table 3:** PR and clinical and ultrasound features in Group II from the training set

Clinical and ultrasound features	Group II		Univariate analysis		Multivariate analysis	
	PR− (ER+, HER2−) (N = 62)	PR+ (ER+, HER2−) (N = 636)	*X*^*2*^ Value	P value	OR (95%CI)	P value
Age (year)			7.747	0.005*		0.007*
≤ 50	18 (29.0)	302 (47.5)			2.204 (1.238–3.924)	
> 50	44 (71.0)	334 (52.5)			1	
Palpable ALN			0.345	0.557		
No	51 (82.3)	541 (85.1)				
Yes	11 (17.7)	95 (14.9)				
US ALN			1.431	0.232		
No	38 (61.3)	437 (68.7)				
Yes	24 (38.7)	199 (31.3)				
Breast cancer family history			0.001	0.974		
No	60 (96.8)	615 (96.7)				
Yes	2 (3.2)	21 (3.3)				
Menopause			6.158	0.013*		
No	20 (32.3)	310 (48.7)				
Yes	42 (67.7)	326 (51.3)				
BMI (kg/m^2^)			1.391	0.499		
< 18	3 (4.8)	15 (2.4)				
18–24	33 (53.2)	351 (55.2)				
> 24	26 (42.0)	270 (42.4)				
Tumor size (mm)			0.002	0.964		
≤ 20	14 (22.6)	142 (22.3)				
> 20	48 (77.4)	494 (77.7)				
Shape			1.664	0.435		
Oval	1 (1.6)	29 (4.6)				
Round	4 (6.5)	28 (4.4)				
Irregular	57 (91.9)	579 (91.0)				
Orientation			0.853	0.356		
Parallel	34 (54.8)	387 (60.8)				
Not parallel	28 (45.2)	249 (39.2)				
Margin			0.140	0.708		
Circumscribed	1 (1.6)	15 (2.4)				
Not circumscribed	61 (98.4)	621 (97.6)				
Lesion boundary			0.007	0.934		
Abrupt	37 (59.7)	383 (60.2)				
Halo	25 (40.3)	253 (39.8)				
Echo pattern			3.643	0.303		
Hypoechoic	59 (95.2)	554 (87.1)				
Isoechoic	1 (1.6)	28 (4.4)				
Complex	2 (3.2)	43 (6.8)				
Hyperechoic	0 (0.0)	11 (1.7)				
Posterior acoustic features						0.039*
No features	14 (22.6)	213 (33.5)	8.951	0.03*	1	
Enhancement	26 (41.9)	158 (24.8)			0.418 (0.211–0.830)	0.013*
Shadowing	20 (32.3)	246 (38.7)			0.876 (0.430–1.785)	0.715
Combined	2 (3.2)	19 (3.0)			0.453 (0.115–2.632)	0.453
Calcification			0.032	0.859		
No	33 (53.2)	331 (52.0)				
Yes	29 (46.8)	305 (48.0)				
Vascular degree			0.960	0.811		
0	9 (14.5)	99 (15.6)				
I	9 (14.5)	110 (17.3)				
II	27 (43.6)	285 (44.8)				
III	17 (27.4)	142 (22.3)				

*ER* estrogen receptor, *PR* progesterone receptor, *HER2* human epidermal growth factor receptor-2, *BMI* body mass index, *ALN* axillary lymph node, *US* ultrasound

In Group III, palpable ALN, US ALN, calcifications, shape, and posterior acoustic features were related to HER2 status in the univariate analysis (p < 0.05). Palpable ALN; round, irregular shape; and calcifications were independent predictors of HER2+ (OR 2.319, 95% CI 1.381–3.895 p = 0.001; OR 7.491, 95% CI 1.715–32.724, p = 0.007; OR 3.786, 95% CI 1.369–10.470, p = 0.010; OR 3.346, 95% CI 2.051–5.459, p < 0.001, respectively). In breast cancers, round and irregular shapes were 7.491 and 3.786 times more likely to express HER2+ than oval shapes, respectively; the presence of calcifications was 3.346 times more prone to express HER2+ than those without calcifications; and the presence of palpable ALN was 2.319 times more likely to express HER2+ than those without palpable ALN (Table 4).

**Table 4:** HER2 and clinical and ultrasound features in Group III from the training set

Clinical and ultrasound features	Group III		Univariate analysis		Multivariate analysis	
	HER2− (ER−, PR−) (N = 194)	HER2+ (ER−, PR−) (N = 138)	*X*^*2*^ Value	*P* value	OR (95%CI)	*P* value
Age (year)			2.161	0.142		
≤ 50	83 (42.8)	48 (34.8)				
> 50	111 (57.2)	90 (65.2)				
Palpable ALN			7.429	0.006*		0.001*
No	149 (76.8)	87 (63.0)			1	
Yes	45 (23.2)	51 (37.0)			2.319 (1.381–3.895)	
US ALN			5.922	0.015*		
No	105 (54.1)	56 (40.6)				
Yes	89 (45.9)	82 (59.4)				
Breast cancer family history			1.016	0.313		
No	184 (94.8)	134 (97.1)				
Yes	10 (5.2)	4 (2.9)				
Menopause			2.442	0.118		
No	84 (43.3)	48 (34.8)				
Yes	110 (56.7)	90 (65.2)				
BMI (kg/m^2^)			1.075	0.584		
< 18	3 (1.5)	2 (1.4)				
18–24	119 (61.4)	77 (55.8)				
> 24	72 (37.1)	59 (42.8)				
Tumor size (mm)			0.180	0.671		
≤ 20	29 (14.9)	23 (16.7)				
> 20	165 (85.1)	115 (83.3)				
Shape			10.710	0.005*		0.015*
Oval	28 (14.4)	5 (3.6)			1	
Round	7 (3.6)	7 (5.1)			7.491 (1.715–32.724)	0.007*
Irregular	159 (82.0)	126 (91.3)			3.786 (1.369–10.470)	0.010*
Orientation			0.035	0.851		
Parallel	126 (64.9)	91 (65.9)				
Not parallel	68 (35.1)	47 (34.1)				
Margin			0.032	0.859		
Circumscribed	5 (2.6)	4 (2.9)				
Not circumscribed	189 (97.4)	134 (97.1)				
Boundary			0.251	0.616		
Abrupt	140 (72.2)	103 (74.6)				
Halo	54 (27.8)	35 (25.4)				
Echo pattern			4.203	0.240		
Hypoechoic	175 (90.2)	129 (93.5)				
Isoechoic	2 (1.0)	2 (1.4)				
Complex	15 (7.8)	4 (2.9)				
Hyperechoic	2 (1.0)	3 (2.2)				
Posterior acoustic features			9.179	0.027*		
No	47 (24.2)	35 (25.4)				
Enhancement	106 (54.7)	56 (40.6)				
Shadowing	34 (17.5)	42 (30.4)				
Combined	7 (3.6)	5 (3.6)				
Calcifications			24.579	< 0.001*		< 0.001*
No	111 (57.2)	41 (29.7)			1	
Yes	83 (42.8)	97 (70.3)			3.346 (2.051–5.459)	
Vascular degree			4.973	0.174		
0	27 (13.9)	12 (8.7)				
I	25 (12.9)	23 (16.7)				
II	86 (44.3)	72 (52.2)				
III	56 (28.9)	31 (22.4)				

*ER* estrogen receptor, *PR* progesterone receptor, *HER2* human epidermal growth factor receptor-2, *BMI* body mass index, *ALN* axillary lymph node, *US* ultrasound

All details are illustrated in Tables 2, 3 and 4.

### Performance of prediction model for the HER2+ subtype (non-luminal) in training and test sets

**Fig. 2:**
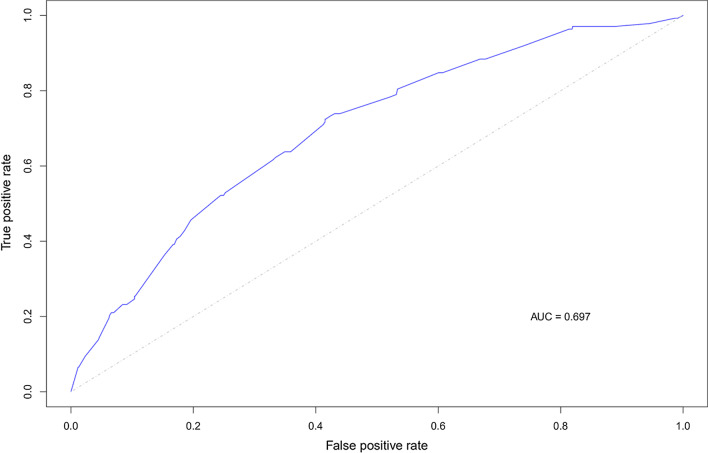
The receiver operating characteristic curve of the predictive model for the HER2 positive subtype in the training set. This figure demonstrates the predictive ability of the model combing clinical and ultrasound features for HER2+ subtype with an AUC of 0.697 in the training set. *HER2* human epidermal growth factor receptor-2, *AUC* area under the receiver operating characteristic curve

**Fig. 3:**
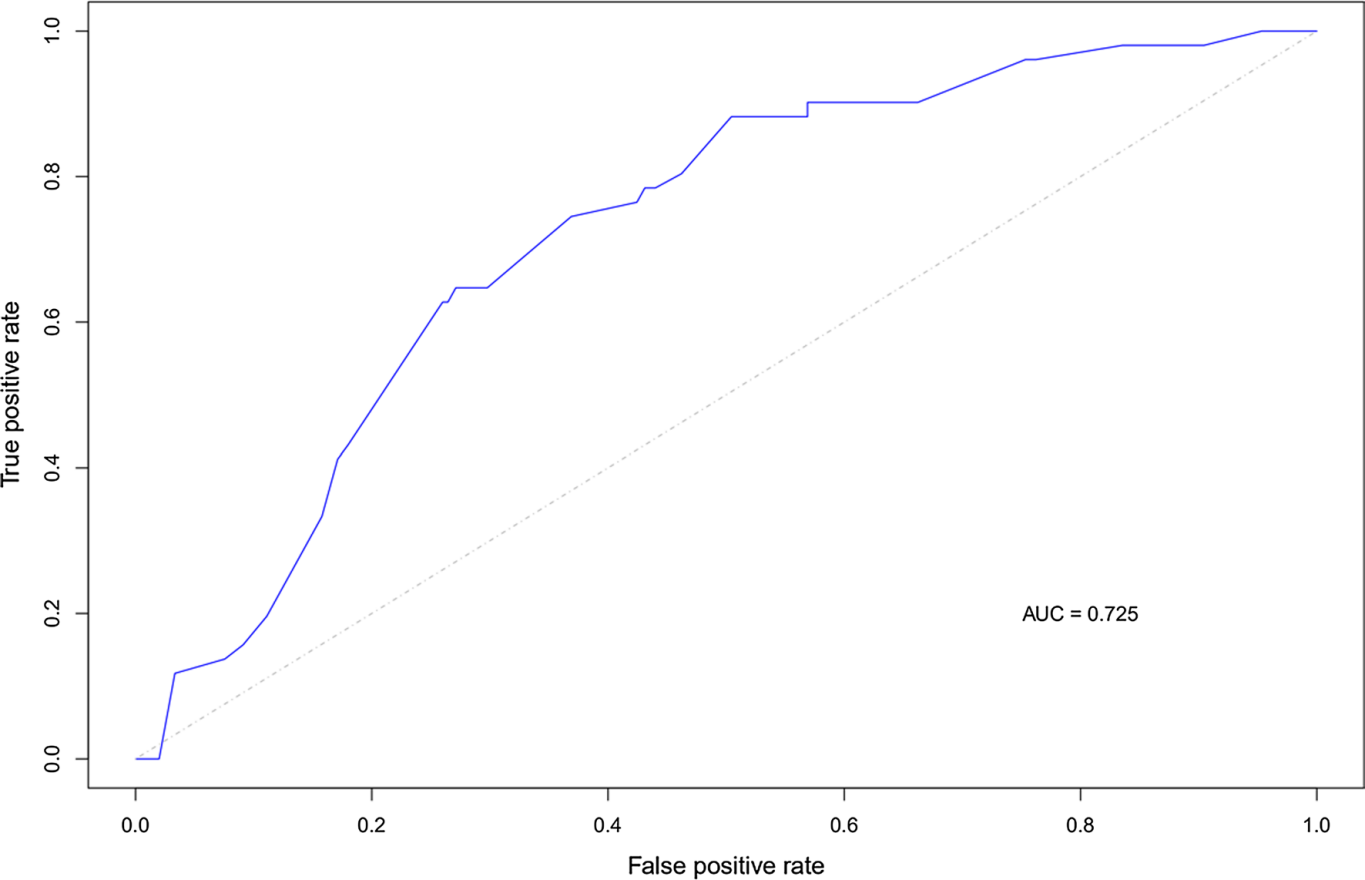
The receiver operating characteristic curve of the predictive model for the HER2 positive subtype in the test set. This figure shows that the model with combined clinical and ultrasound features had moderate predictive power for HER2+ subtype, with an AUC of 0.725 in the test set. *HER2* human epidermal growth factor receptor-2, *AUC* area under the receiver operating characteristic curve

The HER2+ subtype accounted for 138 cases (11.8%) in the training set and 51 cases (10.1%) in the test set. Univariate or multivariate logistic regression analyses of Groups I-III were performed to obtain the relevant features of ER, PR, HER2 (i.e. age, palpable ALN, posterior acoustic features, calcifications, and shape), and to develop a model to predict the HER2+ subtype. The diagnostic efficacy of the model to predict the HER2+ subtype in the training set was AUC 0.697, ACC 60.14%, SENS 72.46%, SPEC 58.49%; and the best cutoff was 0.1028786 (Fig. 2). The efficacy of the model in the test set was AUC 0.725, ACC 72.06%, SENS 64.71%, SPEC 72.89%; and the best cutoff was 0.1321628 (Fig. 3).

## Discussion

Preoperative prediction of biomarkers and breast cancer subtypes can improve the efficiency of the treatment process. It remains unknown that the relationship between clinical and US features and the remaining biomarker, when maintain two of ER, PR, and HER2 in same status. After the matching analysis, the results of this study suggested that, regarding the US features of breast cancers, PR status was mainly reflected by the posterior acoustic features, and HER2 status by the tumor shape and presence of calcifications. None of the US features were found to be independent predictors of ER status when both PR and HER2 were known negative. This study established a predictive model with moderate diagnostic power for predicting the HER2+ subtype.

Biomarker status and molecular subtypes play an important role in the clinical management, outcome and prognosis. For example, ER+/HER2− breast cancer has a better prognosis and requires only endocrine therapy, while the HER2+ subtype requires chemotherapy. The relationship between US features and biomarkers of breast cancer was investigated to better distinguish molecular subtypes before surgery. In previous studies of ER+/ER− breast cancer (regardless of PR and HER2 status), Kim et al. [23] suggested that hypoechoic and complex echo patterns were significantly related to ER and PR negativity; the study by Xu et al. showed an association between ER and PR positivity and echo halo [15]. Inconsistent with previous studies [15, 23], we found no US feature to be an independent predictor of ER status in Group I. However, although tumor's shape did not correlate with ER status in the multivariate regression analysis, but it was still evident that ER- tumors showed a much higher percentage of oval shapes than ER+ tumors (14.4% vs 1.6% in Group I, 9.9% vs 4.1% in the training set). Contrary to a previous study [24], this study suggested that age was not relate with ER in Group I. Additionally, older patients with breast cancer were more frequent in ER+. Although the age structure of the included cases in this study was generally consistent with the study by Zhu et al. [24] (age < 50 years: 39.5% vs 44.8%), the proportion of ER + of both tumors and younger patients (age < 50 years) in the training set was relatively lower in our study (71.5% vs 78.4%, 74.5% vs 80.3%). This may be the reason for the different results.

In Group II, younger age was an independent predictor PR+ (p < 0.005), consistent with the study by Zhu et al. [24]. Besides, this study suggested that younger patients were approximately twice more likely than older patients to express PR+. Tumors with higher histological grade have faster cell proliferation, increased cell and necrotic components, less fibrous tissue, and increased tissue structure uniformity [25]. Considering these characteristics, sound waves can penetrate the tumor to form a posterior echo enhancement without excessive reflection or attenuation. However, previous studies [23, 24, 26–28] have suggested that PR+ breast cancers were more frequently associated with low degrees of malignancy and low histological grades, and Xu et al. indicated directly that the internal necrosis was related to PR negativity. In the training set, 67.6% (507/739) of PR+ lesions were histological grade I/II. Therefore, it’s reasonable that posterior enhancement was negatively correlated with PR+, and that our findings indicated that tumors with posterior enhancement were about 0.4 times more likely to express PR+ than tumors with no change in posterior echogenicity.

HER2 receptors are located in the cell membrane which are involved in the transmission of signals that control normal cell growth and differentiation [29, 30]. HER2 overexpression plays a vital role in tumor transformation and tumorigenesis [29]. In Group III, palpable ALN, shape (round, irregular), and calcifications were independent predictors of HER2+. In previous studies, the presence of calcifications on US or mammography were related to HER2+ [23, 31], which mainly manifests as pleomorphic and branching calcifications on mammography [31]. Several studies [15, 23] suggested that tumor shape was not related to HER2 status; in contrast, our findings suggested that round and irregular shapes were more than two and seven times more likely to appear HER2 positive than oval tumors, respectively. This may only be the relevant in studies that explore the related features of HER2 status in controlled groups. The HER2+ subtype are prone to ALN metastasis (approximately 60% [32]), so it is understandable that our study found that palpable ALN were significantly associated with HER2+.

The HER2+ subtype has a high degree of malignancy and the main pathological type is invasive ductal carcinoma. This study attempted to predict the HER2+ subtype on the basis of the independent predictors of three biomarkers (i.e. age, palpable ALN, posterior acoustic features, calcifications, and shape). Some studies have also shown that the HER2+ subtype was associated with posterior acoustic features, calcifications and age [2, 16, 17]. The diagnostic efficacy of the model in this study was AUC 0.697 in the training set and AUC 0.725 in the test set. To our best knowledge, regarding aspects on predicting breast cancer subtypes based on conventional US feature models, only the study by Zhang et al. [2] was found to have 87.9% accuracy in predicting the HER2+ subtypes using an ensemble decision method based on clinical and US features. Although the present model had inferior performance, however, the two models defined HER2+ subtypes differently, with the former having a 10% cutoff for ER and PR positivity compared with the currently widely used cutoff of 1%. Therefore, the results of this study may be more in line with the current clinical situation. Besides, the diagnostic efficiency of our model was moderate, suggesting the feasibility of predicting breast cancer subtypes based on the related features of biomarkers and providing an alternative modeling idea for predicting subtypes.

This study has certain limitations. First, it was a retrospective study and the US diagnosis was subjective. However, all enrolled cases met uniform imaging standards and had multiple US images to ensure maximum integrity of US features of breast lesions. Additionally, two radiologists independently reviewed the US images, which reduced the subjectivity to a certain extent. Second, despite the considerable size of the data, they were obtained only from a single center. Therefore, data of breast cancer patients from other centers are needed to increase data objectivity. Third, no new image analysis methods were performed, including radiomics or deep learning. The US images in this study were derived from funded projects and have unified image acquisition standards. Thus, they are suitable for image analysis using radiomics to study the relationship between radiomics features and breast cancer subtypes or biomarkers in breast cancer. Although some research in this area [33, 34] has been conducted, some areas could be still improved. However, it is worth noting that data is often affected by uncertainty or inaccuracy. Therefore, it would be necessary to use a fuzzy prediction technique proposed by M Cacciola et al. [35]. We will include this as part of our research in the future.

## Conclusions

Our research suggested that PR status was related to posterior acoustic features, and HER2 status to shape and calcifications. These findings may help non-invasively predict the HER2+ subtype and the status of the biomarkers, and provide an alternative modeling idea for predicting subtypes. Perhaps future studies on the correlation between the expression status of ER, PR, and HER2 and imaging features could consider the influence of biomarkers on each other and might try to change the approach of exploration. In summary, the results could help in formulating an initial impression and treatment plan prior to surgery.

## Data Availability

The datasets used and/or analysed during the current study are available from the corresponding author on reasonable request.

## Abbreviations

ER: Estrogen receptor
PR: Progesterone receptor
HER-2: Human epidermal growth factor receptor-2
US: Ultrasound
IHC: Immunohistochemistry
FISH: Fluorescent in-situ hybridization
CISH: Chromogenic in-situ hybridization
ALN: Axillary lymph node
BMI: Body mass index
AUC: Area under the receiver operating characteristic curve
ACC: Accuracy
SENS: Sensitivity
SPEC: Specificity
Negative: −
Positive: +
